# Supplementary material for: Construction of Reinforced Self-Cleaning and Efficient Photothermal PDMS@GDY@Cu Sponges toward Anticorrosion and Antibacterial Applications
Source: Nanomaterials (Basel). 2023 Aug 20;13(16):2381. doi: 10.3390/nano13162381 (PMC10459430; doi:10.3390/nano13162381)
Supplement: Supplementary file 1 [file nanomaterials-13-02381-s001.zip › nanomaterials-2550362-supplementary.docx]

**Supporting information for**

Construction of Reinforced Self-Cleaning and Efficient Photothermal PDMS@GDY@Cu Sponges toward Anticorrosion and Antibacterial Applications

Yi Hu ^1^, Junmei Pu ^1^, Yingzi Hu ^1^, You Zi ^1^, Hongyan Chen ^2^, Mengke Wang ^1,^* and Weichun Huang ^1^

1. School of Chemistry and Chemical Engineering, Nantong University, Nantong 226019, China;

2107320023@stmail.ntu.edu.cn (Y.H.); 2208320003@stmail.ntu.edu.cn (J.P.);
2013320447@stmail.ntu.edu.cn (Y.H.); ziyou@ntu.edu.cn (Y.Z.); huangweichun@ntu.edu.cn (W.H.)

^2^ Engineering Training Center, Nantong University, Nantong 226019, China; chenhy@ntu.edu.cn

***** Correspondence: mengkewang@ntu.edu.cn

**Table S1** Each component in the studied samples.

|  | Cu (wt%) | GDY (wt%) | PDMS (wt%) |
| --- | --- | --- | --- |
| Cu | 100 ± 0 | 0 | 0 |
| PDMS@Cu | 78.4 ± 2.1 | 0 | 21.7 ± 2.1 |
| GDY@Cu-1 | 93.8 ± 0.5 | 6.2 ± 0.5 | 0 |
| GDY@Cu-2 | 87.2 ± 0.2 | 12.8 ± 0.2 | 0 |
| PDMS@GDY@Cu-1 | 75.4 ± 0.3 | 5.0 ± 0.5 | 19.6 ± 0.8 |
| PDMS@GDY@Cu-2 | 71.9 ± 0.1 | 10.6 ± 0.2 | 17.6 ± 0.1 |


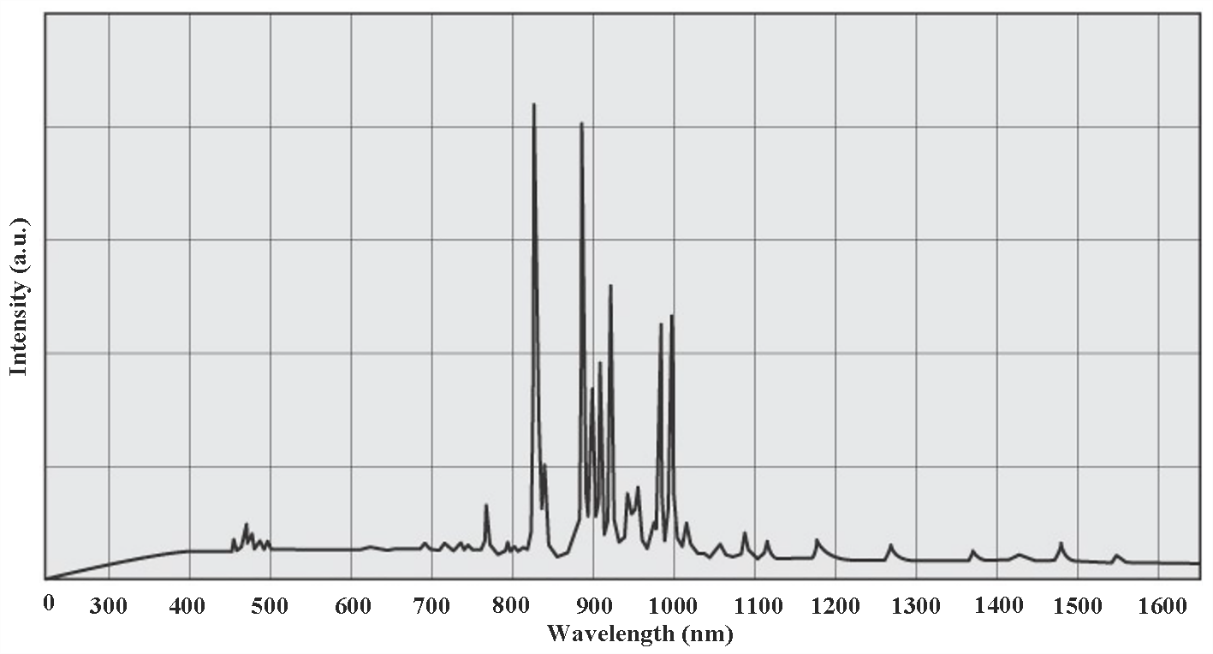


**Figure S1.** Wavelength of the xenon short arc lamp solar simulator.


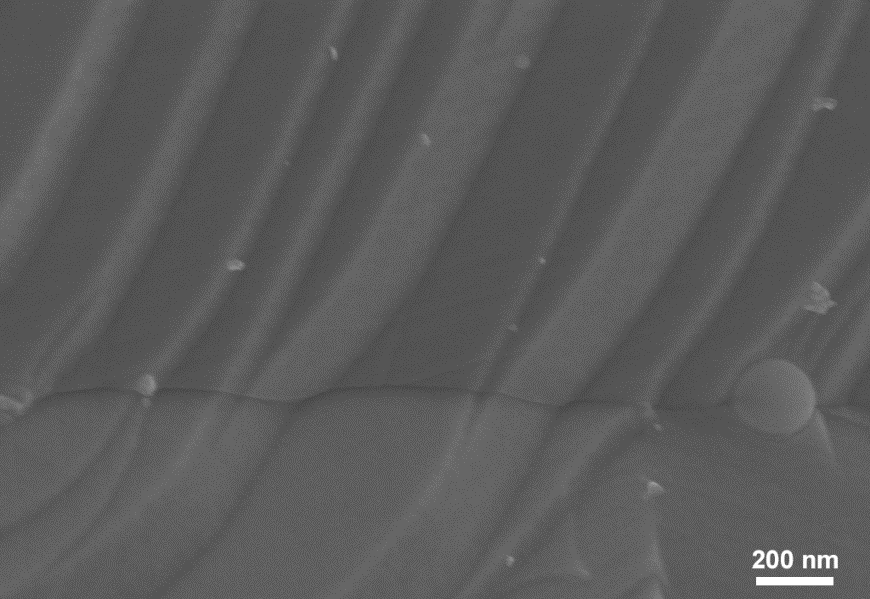


**Figure S2.** SEM image of the pristine Cu sponge.


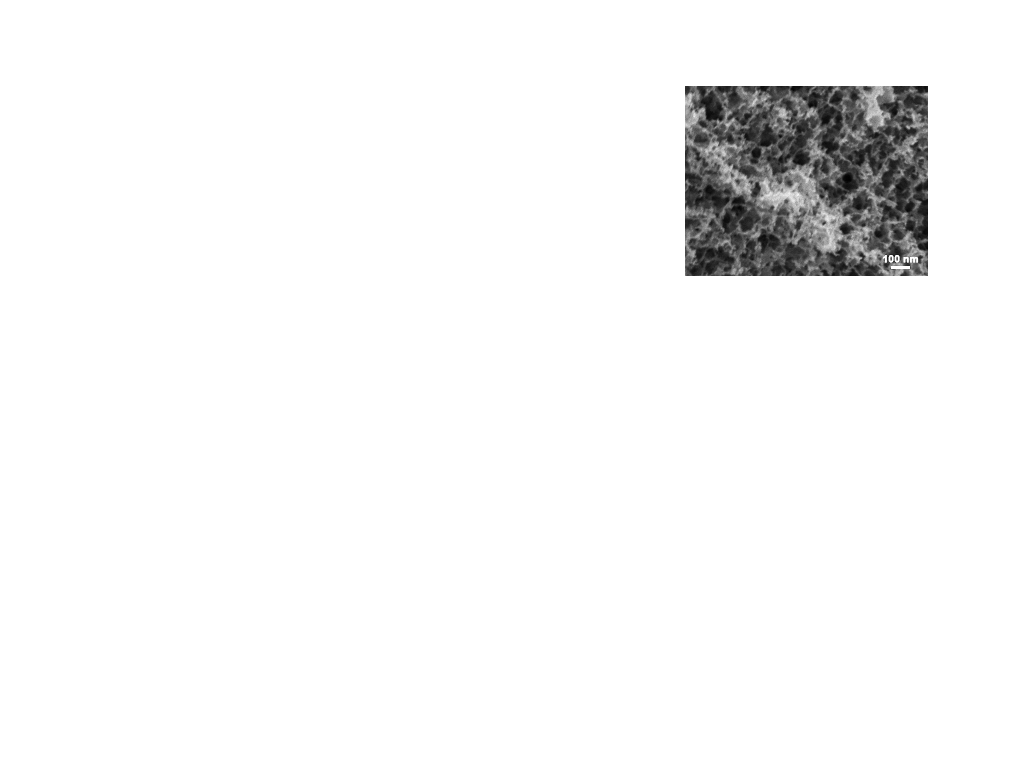


**Figure S3.** SEM image of the GDY@Cu-1.


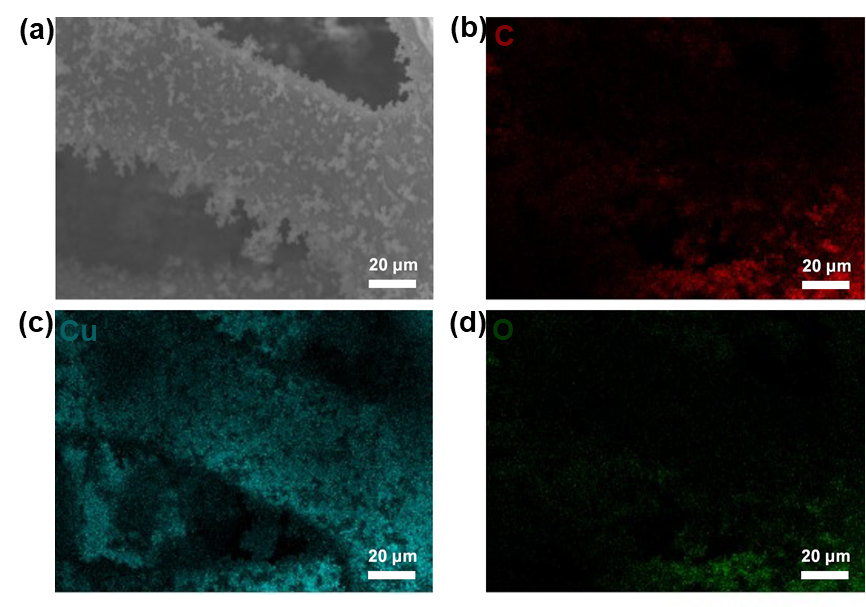


**Figure S4**. (a) SEM image of the GDY@Cu-2 and EDS mapping images of (b) C, (c) Cu, and (d) O elements.


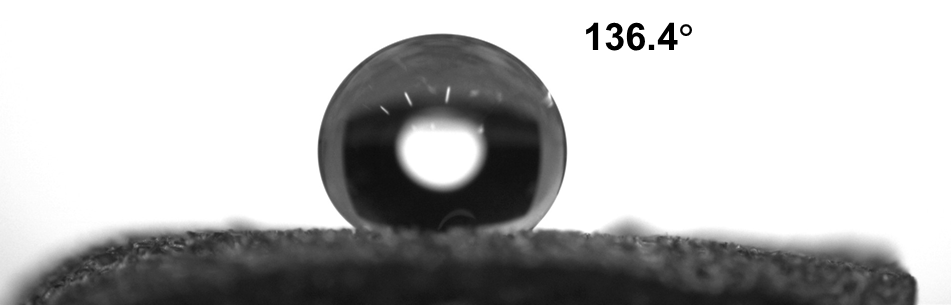


**Figure S5**. Wettability of the PDMS@Cu-1 sponge.


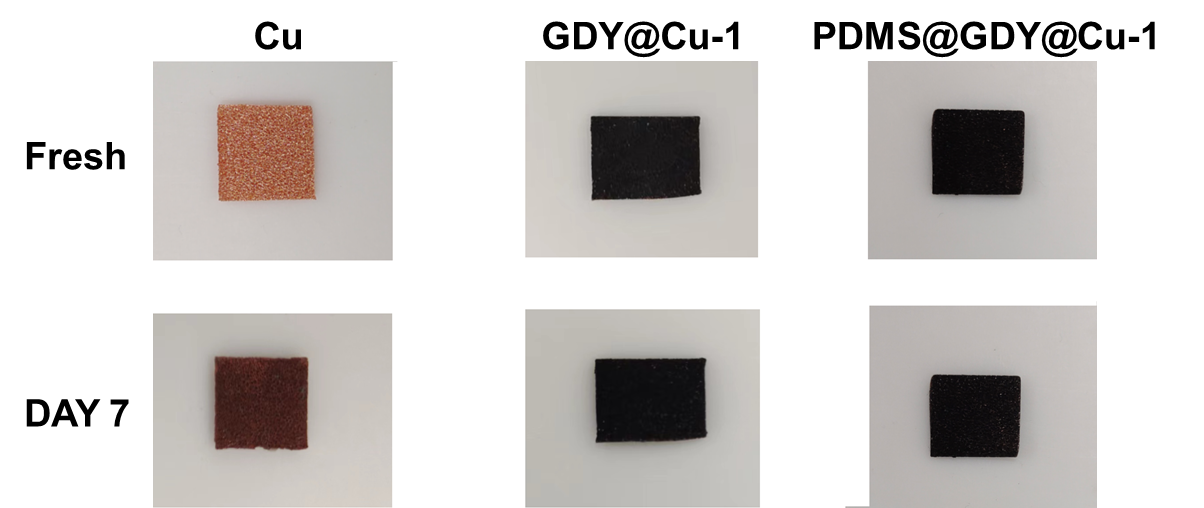


**Figure S6**. Photographs of the samples before and after 7-day EIS characterization.


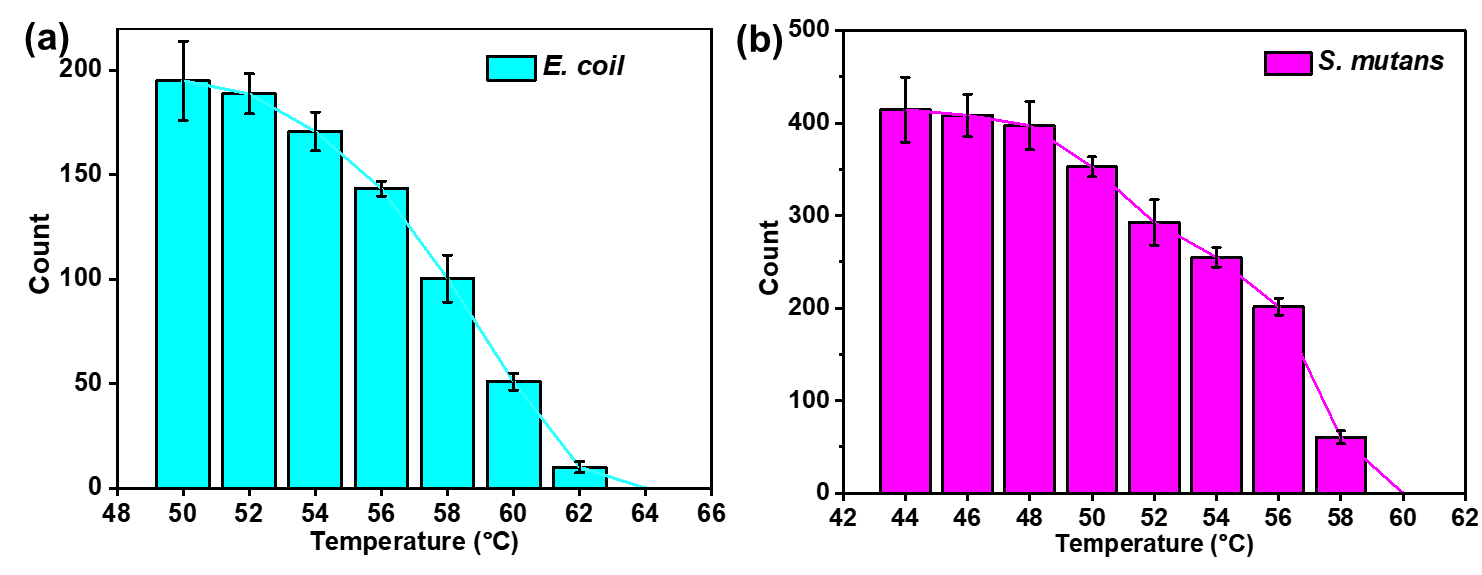


**Figure S7**. Antibacterial activity against (a) *E. coil* and (b) *S. mutans* at different temperatures.


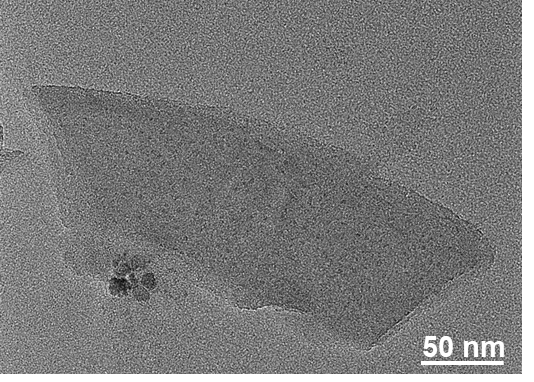


**Figure S8**. TEM image of the GDY obtained by the sonication of PDMS@GDY@Cu-1 after two-week antibacterial experiment.
